# LOCAL USE OF POLLUTING COOKING FUELS AND ADULT COGNITIVE HEALTH

**DOI:** 10.1093/geroni/igae098.4374

**Published:** 2024-12-31

**Authors:** Sneha Mani, Aashish Gupta, Irma Elo

**Affiliations:** Johns Hopkins Bloomberg School of Public Health, Baltimore, Maryland, United States; University of Oxford, Oxford, England, United Kingdom; University of Pennsylvania, Philadelphia, Pennsylvania, United States

## Abstract

Scientific understanding of the relationship between environmental hazards and cognitive health at older ages in low- and middle-income countries (LMICs) is poor. Using data from the Longitudinal Aging Study of India and the World Health Organization’s Survey on Global AGEing and adult health for four LMICs, we examine the association of direct and local exposure to polluting cooking fuels with cognitive health at older ages. We document the negative influence of both: cognitive health is poorer among household members who use polluting fuels and among residents of neighborhoods where the use of polluting fuels is more common. We find that women living in households that use polluting fuels and where the local use of polluting fuels is the highest have the lowest predicted cognitive scores. Conversely, men living in households that do not use polluting fuels and in areas with no local use of polluting fuels have the highest predicted cognitive scores. These associations cannot be explained by accounting for individual or local differences in socioeconomic status. Our findings reveal the substantial direct and indirect influence of polluting fuel use in LMICs and help understand why overall cognitive health may be poor in these settings, especially for women. Additionally, the presence of negative externalities of solid fuel use for cognitive health implies a greater justification for efforts to reduce the use of such fuels.

